# Prothrombin Complex Concentrate in Liver Transplant Surgery: Correction of Therapeutic Anticoagulation and the Coagulopathy of End-Stage Liver Disease: Case Series

**DOI:** 10.3389/fphar.2020.566433

**Published:** 2020-09-08

**Authors:** Scarlett V. Marshall, Jordan Noble, Antolin S. Flores

**Affiliations:** ^1^ Wexner Medical Center, The Ohio State University, Columbus, OH, United States; ^2^ College of Medicine, The Ohio State University, Columbus, OH, United States

**Keywords:** liver transplant anesthesia, liver transplant anesthesiology, prothrombin complex concentrate (PCC), Kcentra, 4-factor prothrombin complex concentrate, end stage liver disease (ESLD), transfusion - alternative strategies

## Abstract

Suggested treatment for active bleeding or invasive procedure prophylaxis has been described in the setting of end-stage liver disease (ESLD) in patients not receiving anticoagulation, and has included fresh frozen plasma (FFP), prothrombin complex concentrates (PCC), platelets, and cryoprecipitate. Today, the therapy for pharmacologically anticoagulated patients with ESLD presenting for liver transplant surgery remains controversial, poorly studied, and physician-dependent. We observed a variety of treatments administered at initiation of liver transplantation to correct acquired coagulopathy at our leading transplant center and present these cases. Three patients receiving preoperative therapeutic anticoagulation with warfarin for acute deep venous thrombosis and/or atrial fibrillation were transfused PCC, FFP, and/or cryoprecipitate for liver or liver-kidney transplant surgery. No thrombotic complications occurred, and one patient required reoperation for hemorrhage. We report data from these cases including estimated blood loss, presence of complications, duration of ICU stay, and length of hospitalization. Perioperative orthotopic liver transplant hematologic management and a review of relevant literature is presented.

## Introduction

Altered coagulation inherent to end stage liver disease (ESLD) can prove difficult to manage in the medical patient, but is particularly challenging in the patient who may urgently undergo liver transplantation (LT). The ESLD population exhibits derangement in traditional coagulation studies often demonstrating a coagulopathic patient, while more modern viscoelastic testing and clinical studies may define a patient as hypercoagulable and at increased risk for thrombosis (Bezinover et al., 2018). Having established a hypercoagulable profile, many subjects who develop thromboses require therapeutic anticoagulation preoperatively. Fresh frozen plasma (FFP) or prothrombin complex concentrates (PCC) as treatment for active bleeding or in preparation for invasive procedure have been described for patients with ESLD not receiving anticoagulation. However, the recommended correction of therapeutic anticoagulation in the ever-more-common anticoagulated patient with ESLD undergoing liver transplantation is not well-described in literature and is varied and physician-dependent in practice (Drebes et al., 2019) as is observed in this case series from our leading transplant center.

PCC is a pooled plasma product, and formulations contain three (II, IX, X) or four (II, VII, IX, X) vitamin K-dependent coagulation factors, proteins C and S, and often heparin (Verbeek et al., 2018; Drebes et al., 2019). PCC factor concentrations are 25 times greater than in FFP, more rapidly and effectively correcting international normalized ratio (INR), with low volume administration that is completed in 10 min (Franichini and Lippi, 2010; Intagliata et al., 2017; Drebes et al., 2019). PCC is easily reconstituted from the lyophilized form and requires no blood group specificity (Cappabiana et al., 2016). PCC likely does not increase the risk of thromboses, and has lower risk of viral transmission and volume overload than FFP (Bezinover et al., 2018; Drebes et al., 2019). Dosing is typically based on INR, however fixed-dosing strategies have been investigated (Leissinger et al., 2008; Dane et al., 2019). KCentra is the only Food and Drug Administration (FDA)-approved four-factor PCC in the United States, and its approved indication is for the reversal of acquired coagulopathy from vitamin K antagonists (VKA) during acute major bleeding or urgent invasive procedure (Sritharan and Triulzi, 2018).

Our tertiary hospital performs over 100 orthotopic LT surgeries each year. Preoperative and postoperative management by surgical and intensive care teams is most often guided by traditional coagulation studies: prothrombin/international normalized ratio (PT/INR), partial thromboplastin time (PTT), platelet count, and fibrinogen level. Intraoperative management includes viscoelastic testing along with cues from patient condition and perceived surgical field bleeding tendency as reported by the surgeon. Balanced transfusion strategies are consistently observed, but therapy to reverse pharmacologic anticoagulation is varied in these three cases of anticoagulation reversal.

The patients presented were treated with PCC monotherapy or in combination with FFP, and one patient was treated with FFP and cryoprecipitate **Table 1**. Each strategy was selected collaboratively by the anesthesiology and surgical teams. The cases are presented to demonstrate varied approaches to treatment of acquired coagulopathy in liver transplant patients. Informed consent was obtained for each case presented.

**Table 1:** Patient characteristics and therapies.

	Case A	Case B	Case C
Anticoagulation	warfarin	warfarin	warfarin
Reason for anticoagulation	DVT	AF	DVT, AF
Preoperative MELD score	21	22	30
Estimated surgical blood loss (L)	0.5	10, 1.5*	8
RBC transfusion required	no	yes	yes
Duration of ICU stay (days)	1	2	2
Duration of hospital stay (days)	5	15	12
Complications	none	Reoperation, acute kidney injury	none

*Estimated blood loss for second operation.

### Case Presentations

#### Case A

A 59-year-old female patient with autoimmune hepatitis-related ESLD was diagnosed with bilateral lower extremity deep venous thromboses (DVT) 1 month prior to liver transplantation and pharmacologically anticoagulated with warfarin with preoperative INR of 2.4. The patient was treated with 1,518 U PCC at initiation of surgery, with resultant normalized extrinsic pathway thromboelastometry (EXTEM) portion of viscoelastic testing and clinically improved surgical field. Traditional coagulation studies were continued for comparison and INR was 1.6 30 min after administration of PCC and 2.4 postoperatively. Intraoperative estimated blood loss (EBL) was 500 ml, and no significant bleeding was evident postoperatively. The patient received no blood products intraoperatively and 2 U of FFP postoperatively. The patient was discharged from the hospital on postoperative day (POD) 5 without postoperative complications.

#### Case B

A 60 year old male patient with nonalcoholic steatohepatitis (NASH) and hepatitis C cirrhosis, and atrial fibrillation (AF) on warfarin presented for LT with preoperative INR of 3. The patient was treated with FFP and cryoprecipitate at initiation of surgery. The following were transfused intraoperatively: 14 U red blood cells (RBC), 16 U FFP, 2 platelet pools, and 4 U cryoprecipitate with an EBL of 10 L. Immediate postoperative INR was 1.3. Due to hemorrhagic shock, the patient returned to the operating room POD 0 where retroperitoneal bleeding was identified, and there was an additional 1.5 L of blood loss. During the subsequent surgery, the patient was transfused 5 U RBC, 2 platelet pools, 3 U FFP, and 4 U cryoprecipitate. Acute kidney injury with peak creatinine of 3.3 recovered, and no other complications were present postoperatively. The patient was discharged on POD 15.

#### Case C

A 67-year-old male patient with NASH cirrhosis, end-stage renal disease (ESRD) on hemodialysis (HD), coronary artery disease (CAD) with stent, who was treated with warfarin for deep venous thrombosis (DVT) and AF with preoperative INR of 2 underwent liver-kidney transplantation. Initial rotational thromboelastometry (ROTEM) identified prolonged clotting time. The patient received 2,112 U PCC and 2 U FFP at the start of surgery. Postoperative INR was 2.6. EBL was 8 L, and 12 U RBC, 7 U FFP, 3 platelets pools, and 5 U cryoprecipitate were transfused. The patient was discharged POD 12 without postoperative complication.

## Discussion

Coagulation in ESLD is dysregulated with alterations occurring at each level of the coagulation pathway (Bezinover et al., 2018; Verbeek et al., 2018; Drebes et al., 2019). There is a decreased concentration of liver-dependent procoagulant factors, while anticoagulant factors proteins C&S and ATIII are also decreased (Bezinover et al., 2018). The reduction in these competing factors is not balanced (Verbeek et al., 2018). Liver-independent factors VIII, vWF, and plasminogen activator inhibitor 1 all increase. Decreased concentration of ADAMTS-13, which deactivates vWF, leads to more active vWF. Thrombocytopenia is impacted by splenic sequestration of platelets, and platelet function is reduced (Bezinover et al., 2018; Verbeek et al., 2018). Fibrinolytic balance is likewise altered with a decrease in liver-dependent factors and an increase in liver-independent factors. Clots in ESLD patients form more slowly, are less permeable and less readily lysed, which is likely related to oxidation of the fibrinogen molecule (Bezinover et al., 2018; Verbeek et al., 2018). Leakage of portal endotoxin from portal to systemic circulation is known to occur in cirrhotic patients and causes endothelial dysfunction by inflammation and oxidative stress (Verbeek et al., 2018).

Derangement in conventional coagulation studies that measure the intrinsic and especially the extrinsic pathway can be expected in ESLD patients (Bezinover et al., 2018; Verbeek et al., 2018). INR is not accurately reflective of coagulation status in ESLD, but instead is more valuable for its prediction of mortality when used for model for end-stage liver disease (MELD) or Child-Turcotte-Pugh score calculation. Viscoelastic testing provides a clearer picture of coagulation status (Northup and Reutemann, 2018).

The net result of all of these changes to the coagulation system is a precarious balance with little reserve, which may be easily tipped in either direction resulting in thrombosis or excessive hemorrhage. Thus, patients with ESLD undergoing LT are at increased risk for bleeding and thromboembolic events (Bezinover et al., 2018). Spontaneous bleeding is uncommon, and bleeding events are more likely to be procedure-related (Verbeek et al., 2018; Drebes et al., 2019). Due to decreased hemostatic reserve, surgical bleeding is more likely to result in decompensation. Intraoperative transfusion of blood products is predictive of survival after LT, and the need for transfusions during LT has declined over time (Bezinover et al., 2018; Verbeek et al., 2018).

### Thrombotic Risk in Chronic Liver Disease

Chronic liver disease (CLD) patients are at greater risk for arterial and venous thromboembolic (VTE) events. Hospitalized CLD patients’ risk for VTE is 0.5–8.2% (Verbeek et al., 2018). Post-LT thrombotic complications are also common with VTE discovered in 5–10%, portal vein thrombosis (PVT) in 2%, and hepatic artery thrombosis (HAT) in 6% of patients. Patients with NASH cirrhosis have higher risk for development of pulmonary embolism (PE), DVT, and PVT, while those with autoimmune disease have greater risk for PVT with higher levels of fibrinogen and tissue factor identified postoperatively. African Americans have greater risk for VTE but lower risk PVT. Hypercoagulability is evident in each phase of transplant care, and does not resolve immediately after transplant (Verbeek et al., 2018).

### Venous Thromboembolic Prophylaxis in End Stage Liver Disease

Prophylactic anticoagulation to prevent DVT in hospitalized patients with ESLD is often provided with heparin or low-molecular-weight-heparin (LMWH), but activity of these medications is difficult to monitor, with an expected baseline elevation of PTT. Forty-four percent of hospitalized patients with CLD receive pharmacologic prophylaxis, a statistic that is increasing. Prophylaxis appears to be safe, though literature in this specific population is limited. Among postoperative patients receiving DVT prophylaxis who have undergone resection for hepatocellular carcinoma, the preoperative presence of esophageal varices (EV) has been identified as the only risk factor for bleeding complications. It is recommended that hospitalized patients with CLD receive DVT prophylaxis with heparin or LMWH if there is no contraindication, and alternatively sequential compression devices should be used (Verbeek et al., 2018).

### Therapeutic Anticoagulation in Chronic Liver Disease

Therapeutic anticoagulation for VTE in ESLD patients has been described with the use of several classes of anticoagulant. VKAs may be used but have a narrow therapeutic index with considerations including diet, monitoring, and underlying disease. Pharmacokinetics are altered in CLD patients due to changes in absorption, volume of distribution, and decreased protein binding. Baseline INR elevation is often present and there may be inter-laboratory variability of testing. Additionally, increasing the INR impacts the MELD score (Verbeek et al., 2018).

Therapeutic anticoagulation in ESLD with direct-acting oral anticoagulants (DOAC) has been limitedly described, and larger trials of these medications excluded cirrhotic patients (Verbeek et al., 2018). Intagliata et al. demonstrated similar risk for bleeding events among 39 cirrhotic patients treated for 3 years with DOAC or warfarin. No specific risk factors for bleeding events were identified among these groups (Intagliata et al., 2016).

Factor Xa inhibitors show similar rates of complication to controls in Child-Turcotte-Pugh classes A and B, data which may not be applicable in the transplant population (Verbeek et al., 2018). Reversal of this class with PCC and other products is not well-studied. Dialysis or idarucizumab, which has been described as safely administered during LT, may be considered in the case of dabigatran (Intagliata et al., 2017; Verbeek et al., 2018). Andexanet is approved by the FDA for reversal of rivaroxaban and apixaban but was not studied in cirrhotic patients (Intagliata et al., 2017).

Postoperative HAT prophylaxis with nonsteroidal anti-inflammatory drugs (NSAIDs) may be considered with consideration for risk of bleeding from EV and the risk of acute renal failure. NSAIDs appear to be safe therapy in ESLD patients with EV after coronary artery stenting (Verbeek et al., 2018).

There remains no agent of choice for anticoagulation and its selection should be determined on an individual basis. Conservative dosing is recommended with any anticoagulant therapy in the ESLD population (Verbeek et al., 2018).

### Treatment of End Stage Liver Disease Coagulopathy

The current standard of care to remedy coagulopathy in ESLD patients who are not receiving systemic anticoagulation and who have active bleeding or planned invasive procedure is FFP, with suggested volume of at least 15 ml/kg (Northup and Reutemann, 2018; Drebes et al., 2019). PCC may alternatively be considered. In all clinical practice, PCC is used most frequently for reversal of VKAs, which cause major bleeding complications in 1.1–1.5% of patients annually (Franichini and Lippi, 2010).

Most studies show no clinically meaningful reduction in bleeding risk to preprocedural transfusion of FFP and rarely is INR goal met in non-anticoagulated patients. Large volume, risk for transfusion-related acute lung injury (TRALI), and volume overload have been reported with FFP administration. Northup et al. do not recommend the use of FFP use for INR reduction preprocedure in ESLD patients due to the risk for life-threatening complications of transfusion and the lack of supportive data for clinically reduced bleeding (Northup and Reutemann, 2018).

### Prothrombin Complex Concentrate for Reversal of Anticoagulation in Other Surgical Populations

Preprocedure anticoagulation with warfarin is well-described in patients with atrial fibrillation undergoing heart transplantation. Both FFP and PCC have been used for corrective therapy (Salerno et al., 2017). Kantorovich et al. demonstrated in a retrospective cohort study that used low-dose PCC for preoperative heart transplant warfarin correction with successful reduction of INR and transfusion of FFP, and no greater risk of thromboembolism (Kantorovich et al., 2015).

Arachchillage *et al*. studied 344 patients taking warfarin, rivaroxaban, and apixaban with major bleeding events, most commonly intracranial hemorrhage, who received treatment with PCC. Treatment was considered to be equally successful in 72.5–77.6% of patients from each of the three anticoagulant therapy groups. Thromboembolic events were statistically similar across the groups at 2.5–5.1%. There was no difference in transfusion of other products among the three groups (Arachchillage et al., 2019). Arachchillage also retrospectively evaluated patients undergoing cardiac surgery whose anticoagulation therapy was treated with PCC or FFP and found among the PCC group significantly less cardiac failure related to circulatory overload; no difference was reported in 30 days mortality, kidney injury, or thrombotic events. However, the same study reported a statistically significant increase in blood loss at 24 h post-surgery (1,575 *vs*. 1,213 ml) (Arachchillage et al., 2016).

### Thrombotic Risk of Prothrombin Complex Concentrate

Previously there was concern for risk of thrombotic complications with PCC, however these events appear to be related to high or repeated doses of PCC. Drebes et al. did not show an increased rate of thrombotic complication with the use of slightly less than the recommended lowest dose for reversal of vitamin K antagonist of 25 IU/kg with a median dose of 22 IU/kg in 105 patients given PCC for preprocedure prophylaxis or bleeding event (Drebes et al., 2019). Other acquired coagulopathies have been treated with PCC and are described in the literature. Sritharan et al. performed retrospective study of 213 patients with bleeding events who received PCC for correction of coagulopathy associated from anticoagulation with warfarin (40%), apixaban (21%), rivaroxaban (23%) therapies and the remaining 16% from cirrhosis, disseminated intravascular coagulation (DIC), or antiplatelet medication. There was lower mortality among the coumadin, rivaroxaban, apixaban groups; and no benefit in the cirrhosis, DIC, or antiplatelet groups. They reported a significantly increased risk of DVT and stroke in the cirrhosis group and a moderate increase for the rivaroxaban group (Sritharan and Triulzi, 2018). As mentioned in the above section, Kantorovich and Arachnillage performed separate studies on diverse populations who received PCC and reported no increase in thrombotic events.

Intraoperative intracardiac thrombus (ICT) or PE occurs in 1–6% of LT patients and carries a high mortality, with actual incidence likely greater with increased identification as transesophageal echocardiography use becomes more common (Dalia et al., 2017). Risk factors for intraoperative thrombotic events include preoperative VTE, history of transjugular intrahepatic portosystemic shunt (TIPS) procedure, use of veno-veno bypass, and use of PA catheter or antifibrinolytics (Verbeek et al., 2018). In a retrospective analysis of 30 patients, 10 of whom received PCC during preoperative hospitalization, Misel et al. reported five patients with intraoperative ICT. An association was determined to include preoperative PCC dose within 14 days of surgery, a history of GI bleeding, and possibly preoperative dialysis treatment. The median time for PCC administration before surgery was 4–6.5 days and usually greater than one dose was given (Misel et al., 2018).

The literature available provides reassuring data. PCC appears to be safest in patients with ESLD who are anticoagulated, with the exception of moderately increased thrombotic risk in patients receiving rivaroxaban. Particular attention to risk for thrombotic complication must be considered if treating patients with cirrhosis-related coagulopathy not undergoing LT, or if electing to use PCC during hospitalization in the several days leading to LT.

### Prothrombin Complex Concentrate Use in Liver Transplantation

One year survival after LT is reduced in patients who receive more blood products intraoperatively, though causality is not concluded as many factors contribute to the need for transfusion. Transfusion of blood products has been associated with an increased risk of postoperative infection, respiratory complications, duration of hospital stay, and graft failure. Red blood cell (RBC) and FFP transfusion has been associated with increased age, PT, length of surgery, and blood urea nitrogen (BUN), presence of cirrhosis, or decreased serum albumin concentration. Other factors associated with a greater need for FFP were preoperative encephalopathy and elevated AST. Platelet transfusion is more likely with increased age, length of surgery, BUN, and PT, or decreased serum albumin (Kasraian et al.,).

Villalpando et al. reported that PCC given at the beginning of LT to correct non-acquired ESLD coagulopathy in 39 patients reduced the EBL by about 500 ml and reduced the need for FFP transfusion. Complications were not reported (Leal-Villalpando et al., 2016). In another retrospective cohort study, Srivastava et al. evaluated the utility of PCC as first line therapy in LT coagulopathy. PCC administration was associated with less RBC and FFP transfusion, and less incidence of hemorrhagic complication requiring surgical re-exploration. Groups had the same need for postoperative ventilation, HD, length of stay, occurrence of thrombosis, or thromboembolism. Some significant differences between the two groups’ characteristics were present and those receiving PCC were often in worse health with higher MELD, creatinine, INR, and lower hemoglobin, platelet level, and/or serum fibrinogen level. Propensity matching grouped 60 similar pairs (Srivastava et al., 2018).

### Combination Therapy/Future Direction for Liver Transplantation

A retrospective, observational analysis by Kirchner et al. in Germany, which studied fibrinogen concentrate (FibC) and PCC together as a combined first-line, ROTEM-guided hemostatic therapy for coagulopathy in LT found no increase in the risk of thrombotic or ischemic events. Significantly fewer RBC units were transfused in the factor concentrate group when groups with and without coagulation factor concentrate were compared (Kirchner et al., 2014). A competing study found no difference in transfusion requirement in patients given PCC and FibC during LT (Colavecchia et al., 2017).

## Conclusion

Acquired coagulopathy was treated in three liver transplant patients with PCC monotherapy, in combination with FFP, or alternatively with FFP and cryoprecipitate. The cases were presented to illustrate the present variable therapy utilized for anticoagulated patients undergoing LT.

Both case A and case C received PCC to treat acquired coagulopathy related to warfarin therapy. However, only case A received no blood products intraoperatively. It is important to consider that case C was a more complex liver-kidney transplant surgery, in a patient with additional comorbidities of CAD, ESRD, and AF, though this should not suggest causation.

Transfusion requirement was less for the patient treated with PCC monotherapy, however the surgery was less complex and there were fewer significant comorbidities present at baseline. The only patient to require reoperation in this limited sample was the patient who was treated initially with FFP and cryoprecipitate. Among the patients, acute kidney injury (AKI) was the only reported postoperative complication.

Further clinical trials are warranted to determine safe, optimal therapy for the coagulopathic patient with ESLD urgently undergoing LT, and therapy continues to be varied in clinical practice. PCC appears to be an effective treatment for acquired coagulopathy in patients with ESLD undergoing LT who have received pharmacologic anticoagulation, with little or no apparent increase in rate of complications. In patients with ESLD-related coagulopathy who are not anticoagulated, it may be reasonable to select PCC as first-line therapy. In this case, there must be careful consideration for possible thrombotic risk noted in patients with cirrhosis treated for bleeding events with PCC who were not undergoing LT, and for those hospitalized patients treated several days prior to LT.

## Data Availability Statement

All datasets presented in this study are included in the article/supplementary material.

## Ethics Statement

Written informed consent was obtained from the individual(s) for the publication of any potentially identifiable images or data included in this article.

## Author Contributions

JN and AF contributed to writing and editing of the manuscript. All authors contributed to the article and approved the submitted version.

## Conflict of Interest

The authors declare that the research was conducted in the absence of any commercial or financial relationships that could be construed as a potential conflict of interest.

The handling editor declared a shared affiliation with the authors at time of review.
